# Left circumflex coronary artery from the pulmonary artery in scimitar syndrome

**DOI:** 10.1007/s00247-017-4067-7

**Published:** 2018-03-14

**Authors:** Ilaria Bo, Thomas Semple, Emma Cheasty, Michael B. Rubens, Siew Yen Ho, Michael L. Rigby, Edward D. Nicol

**Affiliations:** 1 Department of Paediatric Cardiology, Royal Brompton Hospital, Sydney Street, London, SW3 6NP UK; 2 Department of Radiology, Royal Brompton Hospital, Sydney Street, London, SW3 6NP UK; 3 National Heart and Lung Institute, Imperial College London, Dovehouse Street, London, SW3 6LR UK; 4 Department of Cardiac Morphology, Royal Brompton Hospital, Sydney Street, London, SW3 6NP UK; 5 Department of Cardiology, Royal Brompton Hospital, Sydney Street, London, SW3 6NP UK

**Keywords:** Angiography, Anomalous left coronary artery from the pulmonary artery, Cardiac computed tomography, Children, Congenital heart disease, Coronary anomaly, Scimitar syndrome

## Abstract

**Background:**

Scimitar syndrome is a rare combination of cardiopulmonary abnormalities found in 1–3 per 1000 live births. Anomalous origin of the left coronary artery from the pulmonary artery (ALCAPA) is only found in 1 in 250–400 congenital heart disease patients.

**Objective:**

We aimed to investigate the incidence of left circumflex ALCAPA within our referral center’s cohort of scimitar syndrome patients.

**Materials and methods:**

A review of medical records, cardiac imaging and operative notes from all patients diagnosed with scimitar syndrome at our center between 1992 and 2016 was undertaken and all imaging reviewed.

**Results:**

Fifty-four patients with scimitar syndrome and imaging were identified. Of these, 3 patients (1 male and 2 female) with ALCAPA were identified, representing an incidence of 5.5% (95% confidence interval [CI] 0–11.67%). In all three cases, the anomalous coronary arising from the pulmonary artery was the left circumflex coronary artery (LCx) and the point of origin was close to the pulmonary arterial bifurcation.

**Conclusion:**

We hypothesize that the prevalence of LCx-ALCAPA, in the setting of scimitar syndrome, may be greater than previously thought. We suggest that any patient with scimitar syndrome, especially with evidence of ischaemia, should be investigated for ALCAPA. Given its noninvasive nature and simultaneous imaging of the lungs, we suggest that cardiovascular CT is the most appropriate first-line investigation for these patients.

## Introduction

Scimitar syndrome (also known as congenital pulmonary venolobar syndrome) is a rare but well-recognized constellation of cardiopulmonary abnormalities comprising partial or total anomalous pulmonary venous return from the right lung to the inferior vena cava, often associated with hypoplasia of the right lung and its branch pulmonary artery, dextrocardia and systemic arterial supply to the right lower lobe. Of patients with scimitar syndrome, 19–31% are reported to have further associated congenital cardiac malformations, the most common associated cardiac abnormalities being atrial septal defects, with a reported coincidence of 70% [1]. Less commonly reported associations include tetralogy of Fallot, ventricular septal defects, aortic coarctation, hypoplastic left heart syndrome, patent ductus arteriosus, cor triatriatum, bicuspid aortic valve and subaortic stenosis [1]. Coronary artery anomalies are reported to be a rare association [2].

**Fig. 1:**
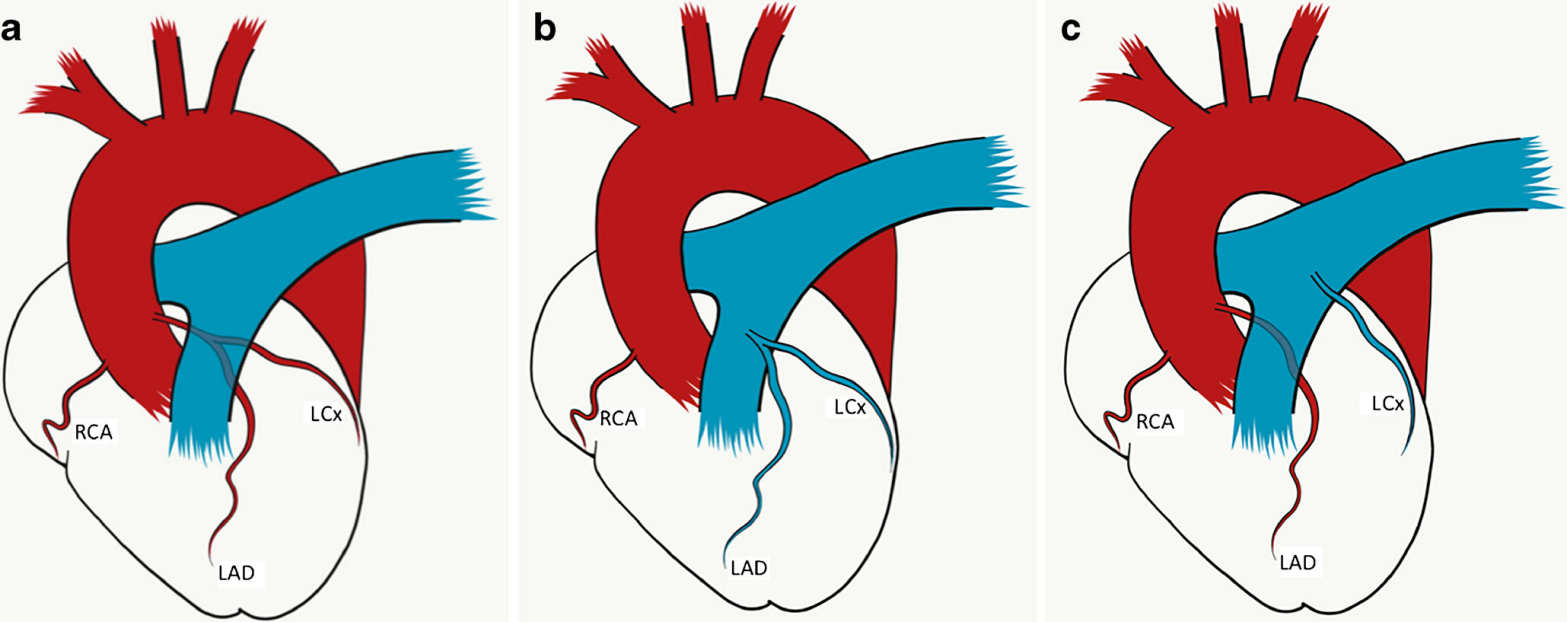
Schematics of relevant normal and abnormal anatomy. **a** Normal coronary artery anatomy. The right coronary artery (RCA) originates from the aorta from the right coronary sinus. The left circumflex (LCx) and left anterior descending (LAD) coronary arteries originate from the aorta from the left coronary sinus as a common trunk – the left mainstem. **b** Anomalous origin of the left coronary artery from the pulmonary artery (ALCAPA). Whilst the right coronary artery (RCA) arises normally, the left mainstem (and therefore both the left anterior descending [LAD] and left circumflex [LCx] coronary arteries) arise from the pulmonary artery and carry deoxygenated blood. **c** Isolated anomalous left circumflex origin from the pulmonary artery. Both the right coronary artery (RCA) and left anterior descending (LAD) coronary arteries arise normally from the aorta, but the left circumflex (LCx) arises from the pulmonary artery. (Artwork from Katriina Nichols)

Following the identification of anomalous origin of the left circumflex coronary artery from the pulmonary artery (LCx-ALCAPA) (Fig. 1) in an infant being worked up for surgical correction of scimitar syndrome in our institution, we performed a Pubmed search that identified very few published case reports of ALCAPA found in association with scimitar syndrome [3, 4]. We therefore undertook a retrospective review of all scimitar syndrome cases investigated at our center to determine the frequency of ALCAPA in patients with scimitar syndrome and the clinical ramifications associated with this combination of congenital malformations.

## Materials and methods

The need for informed consent was waived for this retrospective case review.

A retrospective review of medical records, cardiac imaging (echocardiography, invasive catheter angiography and cardiac CT) and operative notes was undertaken, from all patients diagnosed or referred with scimitar syndrome at our tertiary referral center for paediatric and adult congenital heart surgery between 1992 and 2016.

CT examinations were performed on a 128-slice, dual-source CT scanner (Definition FLASH; Siemens, Erlangen, Germany), manually triggered following the administration of 2 ml/kg of iodinated contrast material (Visipaque 350, GE healthcare AS, Nycoveien 1-2, NO-0401, Oslo, Norway). CT examinations were reviewed by a cardiac radiologist with 30 years of experience or an imaging cardiologist with 15 years of experience.

## Results

Fifty-four patients with scimitar syndrome and imaging +/− operative notes were identified. Of these, 3 patients (1 male and 2 female) with ALCAPA were identified, representing an incidence of 5.5% (95% CI: 0–11.67%). In all three patients, the anomalous coronary arising from the pulmonary artery was the left circumflex coronary artery (LCx) and in all three patients, the point of origin was close to the pulmonary arterial bifurcation. The left anterior descending coronary artery arose from the left coronary sinus. The patients are discussed below and summarized in Table 1.

**Table 1:** Summary of patient details

	Case 1	Case 2	Case 3
Gender	Female	Male	Female
Antenatal diagnosis?	Scimitar – yes ALCAPA – no	Scimitar –yes ALCAPA – no	Scimitar – no ALCAPA – no
Symptoms in infancy	Dyspnoea at 1 week of age	No	Tachypnoea, feeding difficulties and poor weight gain.
Features suggestive of presence of ALCAPA	Initially noted on CT. Left ventricular dyskinesia on echo. Ischaemic electrocardiogram (ECG) changes.	Incidentally noted on catheter angiogram.	Transient ischaemic change during catheter angiogram.
Ischaemic ECG changes	Yes - Posterolateral ischaemic change at 7 weeks of age.	Yes – but only on balloon occlusion.	Yes – but only during catheter angiogram.
Pulmonary artery pressures on invasive catheterisation	Suprasystemic	Normal	Elevated
Treatment of ALCAPA?	Surgical reimplantation	None	Surgical reimplantation (alongside pulmonary vein dilatation, right pulmonary artery reconstruction and atrial septal defect repair).
Outcome	Symptom free and well at 3-year follow-up.	Asymptomatic with no treatment.	Died at 7 months of age.

*ALCAPA* anomalous origin of the left coronary artery from the pulmonary artery

## Patient 1

Patient 1 is a female neonate with an antenatal diagnosis of scimitar syndrome. Dyspnoea during the first week of life prompted referral for cardiovascular CT to assess for extracardiac features, difficult to identify on echocardiography such as the size of the systemic arterial collaterals, stenosis of the scimitar vein, airway compression, etc. CT demonstrated dextrocardia, total anomalous pulmonary venous drainage of the right lung to the inferior vena cava and the presence of two aortopulmonary collaterals arising from the aorta, in close proximity to the coeliac axis, supplying a segment of the right lower lobe. The right pulmonary artery and right lung were severely hypoplastic.

**Fig. 2:**
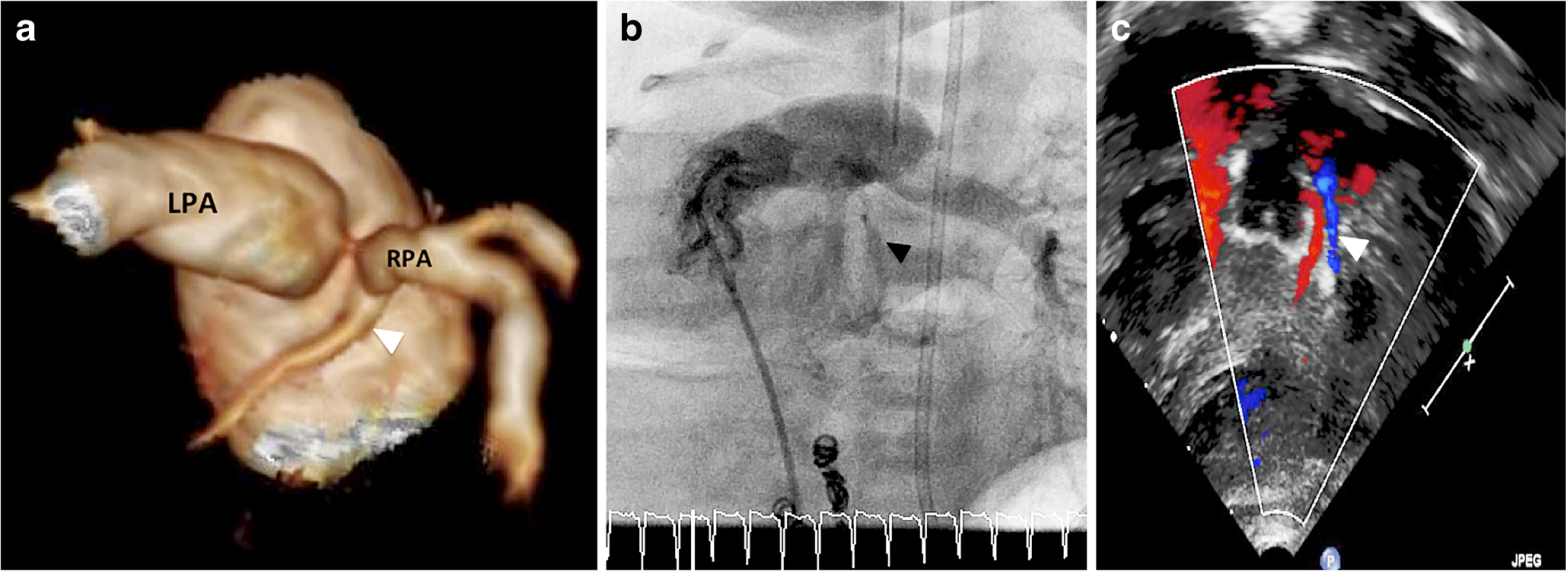
Main imaging findings. **a-c** A female neonate with antenatally diagnosed scimitar syndrome undergoing CT for investigation of unexpected dyspnoea. Posterior projection volume-rendered tomogram (**a**) from an ungated high-pitch CT demonstrates anomalous origin of the left circumflex coronary artery (*arrowhead*) immediately inferior to the origin of the hypoplastic right pulmonary artery (RPA) The left pulmonary artery (LPA) is normal. Invasive catheter angiography (**b**) confirms the anomaly (*arrowhead*). Note the embolisation coils in the systemic arterial collaterals. The anomaly was subsequently also demonstrated via echocardiography (**c**). Retrograde flow was demonstrated in the left circumflex coronary artery (flow in blue marked by an *arrowhead)*. Antegrade flow is demonstrated in red in the adjacent left anterior descending artery

An anomalous LCx coronary artery was demonstrated originating from the bifurcation of the pulmonary trunk, close to the origin of the right pulmonary artery (Fig. 2).

Cardiac catheterization was subsequently performed, which corroborated the CT findings. The pulmonary artery systolic pressure was suprasystemic and the two aortopulmonary collaterals were embolised.

At 7 weeks, echocardiography demonstrated dyskinesia of the left ventricular posterior wall and electrocardiogram appearances were consistent with posterolateral myocardial ischaemia. In view of the evidence of progressive myocardial ischaemia and left ventricular dysfunction, surgical reimplantation of the LCx-ALCAPA was performed at 3 months of age. Within 3 months of the surgery, the girl was symptom-free with normal ventricular function on echo. She remained well at her 3-year follow-up.

## Patient 2

Patient 2 is a male infant with an antenatal diagnosis of scimitar syndrome. He remained asymptomatic during the first 6 months of life.

**Fig. 3:**
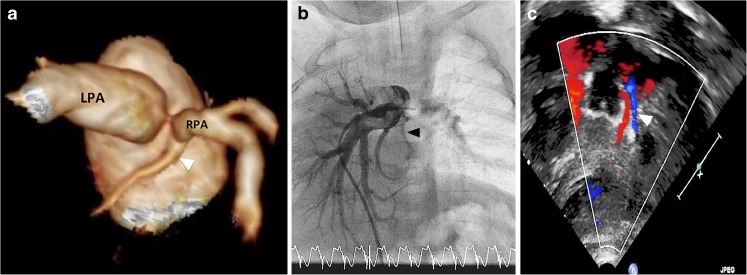
A 6-month-old boy with antenatally diagnosed scimitar syndrome. Angiography following balloon occlusion of the left pulmonary artery demonstrates filling of the anomalous left circumflex coronary (*arrowhead*). Note the ischaemic changes on the electrocardiogram trace during angiography

At 6 months of age, elective cardiac catheterisation was performed, which demonstrated total anomalous pulmonary venous drainage of the right lung to the inferior vena cava, mild right lung hypoplasia and the absence of aortopulmonary collaterals. The pulmonary arterial pressure was normal (25/13 mmHg, mean: 19 mmHg). Selective angiography suggested anomalous origin of the LCx coronary artery from the pulmonary trunk close to the bifurcation, confirmed via balloon occlusion left pulmonary arteriography. Balloon occlusion resulted in transient electrocardiogram changes suggestive of myocardial ischaemia (Fig. 3).

Because the boy remained asymptomatic, no immediate treatment was undertaken and, at 9 months of age, he remained symptom-free with normal ventricular function and no evidence of myocardial ischemia.

## Patient 3

Patient 3 is a female infant transferred to our institution at 10 weeks of age with poor weight gain, feeding difficulties and tachypnoea. Echocardiography demonstrated dextrocardia, a secundum atrial septal defect and a hypoplastic right pulmonary artery. There was normal left ventricular size and function. There was no evidence of ischaemia on her electrocardiogram.

**Fig. 4:**
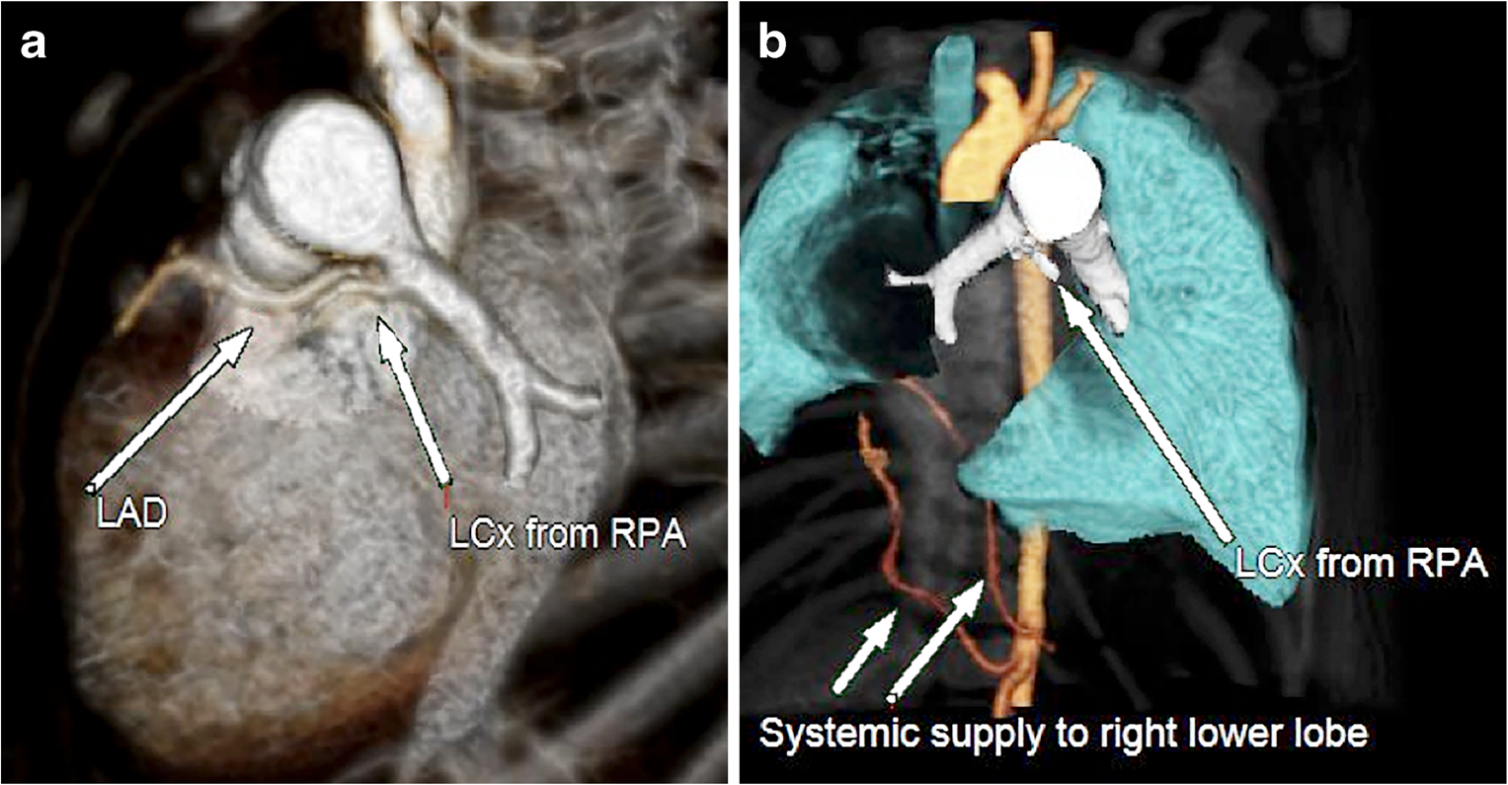
A 10-week-old girl with a postnatal diagnosis of scimitar syndrome. CT was requested to investigate the coronary arteries after noting transient ischaemic electrocardiographic changes during invasive catheter angiography. **a** Posterior oblique volume-rendered tomogram from the CT examination demonstrates (*arrows*) the anomalous left circumflex (LCx) coronary artery arising from the right pulmonary artery origin (RPA) and running adjacent to the left anterior descending (LAD) coronary artery. **b** Volume-rendered tomogram from wider field of view CT images also demonstrates (*arrows*) the two-system arterial collaterals and the hypoplastic right lung. *LAD* left anterior descending coronary artery, *LCx* left circumflex coronary artery, *LPA* left pulmonary artery, *RPA* right pulmonary artery

Initial cardiac catheterization demonstrated her pulmonary artery pressure was elevated (54/14 mmHg, mean: 30 mmHg) with normal systemic systolic pressure. Two small (2-mm diameter) aortopulmonary collaterals were identified. There was moderate hypoplasia of the right pulmonary artery. A hypoplastic anomalous right pulmonary vein connecting to the inferior vena cava was noted on the levophase of the angiogram. The left pulmonary veins appeared stenosed at the junction with the left atrium. Transient ischaemic signs were demonstrated on electrocardiogram prior to and during angiography (Fig. 4).

Cardiovascular CT was requested to further delineate both the cardiac and pulmonary anatomy before intervention. This confirmed the angiographic findings, but it also demonstrated an anomalous LCx-coronary artery arising from the right pulmonary artery, just distal to the pulmonary arterial bifurcation, and a horseshoe lung deformity.

Subsequent selective angiography of the right pulmonary artery, in the catheter laboratory, confirmed the LCx-ALCAPA and the small aortopulmonary collaterals were successfully occluded using embolisation coils.

Despite transcatheter cutting balloon dilatations of the left pulmonary vein stenosis, surgical reimplantation of the LCx coronary artery, reconstruction of the right pulmonary artery and fenestrated atrial septum defect repair, the patient died at 7 months of age from recurrent left pulmonary vein stenosis and resultant severe pulmonary hypertension and right ventricular failure.

## Discussion

ALCAPA is reported to occur in only 1 in 250–400 congenital heart malformations with an overall incidence of approximately 1 in 300,000 live births [5]. The isolated LCx-ALCAPA variant is even less common and has only occasionally been reported in the literature, as either an isolated malformation [6–9] or in association with other anomalies of the aortic and pulmonary trunk (including aortopulmonary window [10], subaortic stenosis and coarctation of the aorta [3, 11, 12]).

Scimitar syndrome is a rare anomaly with an incidence of approximately 1 to 3 per 100,000 live births; however, the true incidence may be higher as many patients are asymptomatic [13]. ALCAPA in conjunction with scimitar syndrome has only been reported in the literature in three patients [2, 3, 14] and isolated LCx-ALCAPA has been described at only one other center [15]. The 5.5% incidence of LCx-ALCAPA in our cohort suggests the association may be underrecognized. Of note, the association of ALCAPA variants and scimitar syndrome has not been highlighted in previous comprehensive literature reviews on scimitar syndrome [1, 16].

Chest radiography and echocardiography remain the first-line imaging investigations in these infants, but will rarely demonstrate direct evidence of the presence of an ALCAPA. Although diagnostic conventional angiography is still widely used in paediatric congenital heart disease, noninvasive assessment via cardiovascular CT is becoming an increasingly attractive preoperative imaging modality in complex congenital heart and lung disease assessment with consistently falling radiation dose and decreased need for general anaesthesia/sedation.

The features of combined scimitar syndrome-ALCAPA on both conventional angiography and cardiovascular CT are surprisingly consistent within our small series. When present and specifically sought, it should, therefore, be possible to confidently identify an ALCAPA in scimitar syndrome patients. Ischaemia, suggested by regional wall motion abnormalities at rest or ischaemic electrocardiogram changes on invasive angiography, should lead to increased suspicion of this dual diagnosis. It should be noted, however, that the absence of ischaemic signs and symptoms does not necessarily exclude the diagnosis because scimitar syndrome is often associated with pulmonary hypertension, and the raised pulmonary pressure may provide sufficient perfusion of the circumflex artery to prevent the development of ischaemia.

**Fig. 5:**
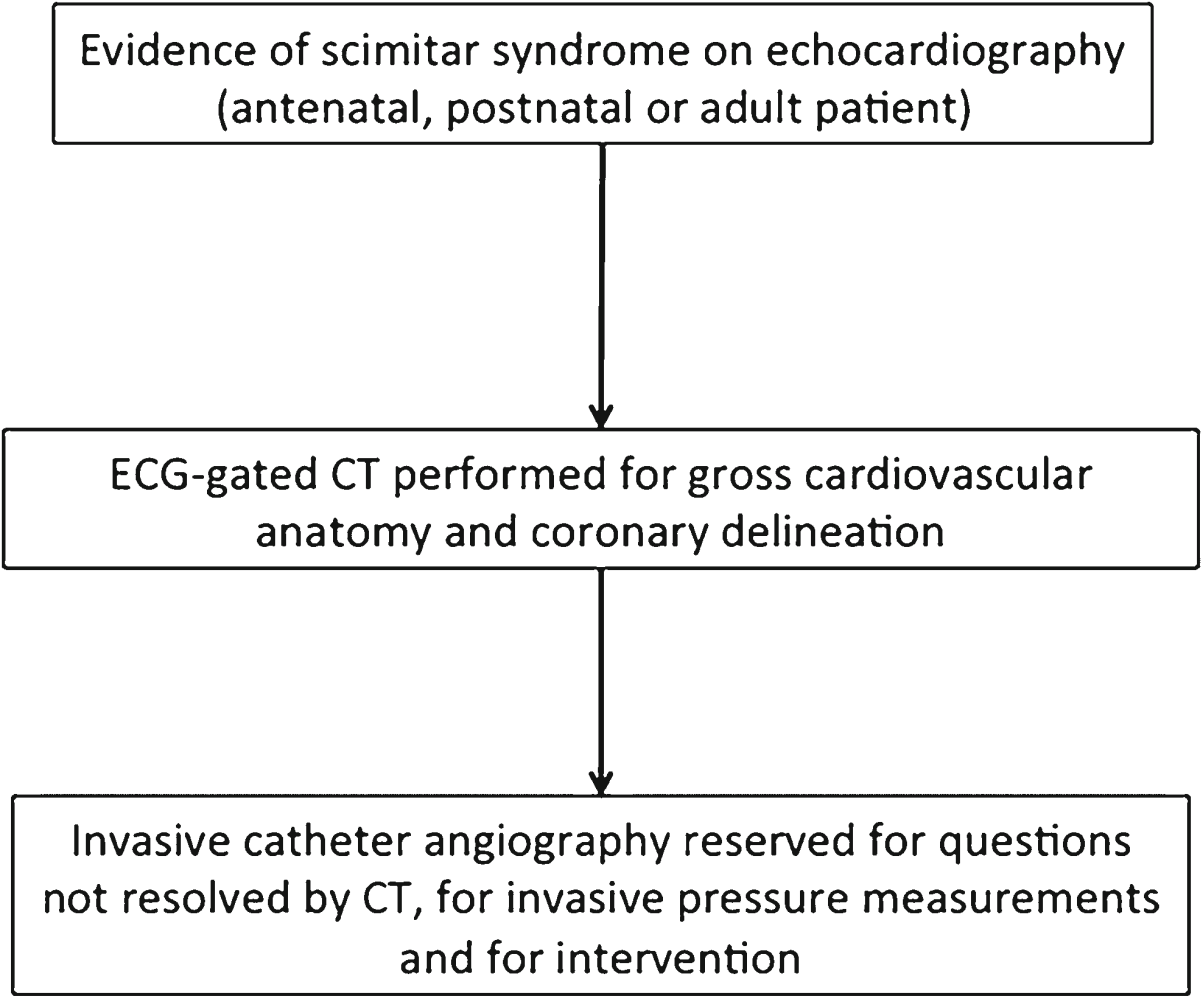
Suggested investigation algorithm following diagnosis or suspicion of scimitar syndrome via echocardiography, *ECG* electrocardiogram

CT, used to assess gross cardiovascular anatomy in complex congenital heart disease, is increasingly performed as non-electrocardiogram-gated, high-pitch spiral or volumetric acquisitions. Our finding of associated coronary anomalies implies it may be worth utilizing a specific electrocardiogram-gated protocol when assessing patients with scimitar syndrome to maximize the chances of adequate coronary visualization regardless of the presence of absence of evidence of ischaemia on electrocardiography (Fig. 5).

## Conclusion

With a high index of suspicion and the advent of low-dose, high-quality cardiovascular CT, used in conjunction with invasive angiography, we believe we have uncovered a clinically important association between two rare clinical entities.

We suggest that any patient presenting with scimitar syndrome should be investigated in a manner that allows the diagnosis of ALCAPA and other associations to be made. Given its noninvasive nature and ability to simultaneously image the lungs, cardiovascular CT is the most appropriate first-line investigation for patients with suspected scimitar syndrome on echocardiography.

## References

[1] Vida VL, Padalino MA, Boccuzzo G, Tarja E (2010). Scimitar syndrome: A European congenital heart surgeons association (ECHSA) multicentric study. Circulation.

[2] Gao YA, Burrows PE, Benson LN (1993). Scimitar syndrome in infancy. J Am Coll Cardiol.

[3] Ilic S, Hercog D, Vucicevic M (2014). Anomalous origin of the left coronary artery from the pulmonary artery, scimitar syndrome, and aortic coarctation. Ann Thorac Surg.

[4] Laux D, Bertail C, Bajolle F (2014). Anomalous left coronary artery connected to the pulmonary artery associated with other cardiac defects: A difficult joint diagnosis. Pediatr Cardiol.

[5] Keith JD, Keith JD, Rowe RD, Vlad P (1978). Diseases of coronary arteries and aorta. Heart disease in infancy and childhood.

[6] Garcia CM, Chandler J, Russell R (1992). Anomalous left circumflex coronary artery from the right pulmonary artery: first adult case report. Am Heart J.

[7] Korosoglou G, Ringwald G, Giannitsis E, Katus HA (2008). Anomalous origin of the left circumflex coronary artery from the pulmonary artery. A very rare congenital anomaly in an adult patient diagnosed by cardiovascular magnetic resonance. J Cardiovasc Magn Reson.

[8] Ott DA, Cooley DA, Pinsky WW, Mullins CE (1978). Anomalous origin of circumflex coronary artery from right pulmonary artery: report of a rare anomaly. J Thorac Cardiovasc Surg.

[9] Bolognesi R, Alfieri O, Tsialtas D, Manca C (2003). Surgical treatment of the left circumflex coronary artery from the pulmonary artery in an adult patient. Ann Thorac Surg.

[10] Chopra PS, Reed WH, Wilson AD, Rao PS (1994). Delayed presentation of anomalous circumflex coronary artery arising from pulmonary artery following repair of aortopulmonary window in infancy. Chest.

[11] Sarioglu T, Kinoglu B, Saltik L, Eroglu A (1997). Anomalous origin of circumflex coronary artery from the right pulmonary artery associated with subaortic stenosis and coarctation of the aorta. Eur J Cardiothorac Surg.

[12] Honey M, Lincoln JC, Osborne MP, de Bono DP (1975). Coarctation of aorta with right aortic arch. Report of surgical correction in 2 cases: one with associated anomalous origin of left circumflex coronary artery from the right pulmonary artery. Br Heart J.

[13] Dupuis C, Charaf LAC, Brevière GM (1992). The ‘adult’ form of the scimitar syndrome. Am J Cardiol.

[14] Böning U, Sauer U, Mocellin R (1983). Anomalous coronary drainage from the pulmonary artery with associated heart and vascular abnormalities. Report on 3 patients and review of the literature. Herz.

[15] Lee T-M, Chen W-J, Chen M-F (1995). Anomalous origin of left circumflex artery in a scimitar syndrome: A case report. Angiology.

[16] Dusenbery SM, Geva T, Seale A (2013). Outcome predictors and implications for management of scimitar syndrome. Am Heart J.

